# κ-Opioid receptor activation attenuates osteoarthritis synovitis by regulating macrophage polarization through the NF-κB pathway: KOR regulates OA synovitis via NF-κB

**DOI:** 10.3724/abbs.2023223

**Published:** 2023-11-28

**Authors:** Yi Shi, Huaqiang Tao, Xueyan Li, Liyuan Zhang, Chunhui Li, Wen Sun, Miao Chu, Kai Chen, Pengfei Zhu, Qiang Wang, Chengyong Gu, Liangliang Wang, Xing Yang, Yuefeng Hao

**Affiliations:** 1 Anesthesiology Department Suzhou Municipal Hospital (North District) Nanjing Medical University Affiliated Suzhou Hospital Suzhou 215000 China; 2 Department of Orthopedics the First Affiliated Hospital of Soochow University Suzhou 215000 China; 3 Department of Orthopedics Changshu Hospital Affiliated to Soochow University First People’s Hospital of Changshu City Changshu 215500 China; 4 Department of Orthopedics the Affiliated Changzhou No. 2 People’s Hospital of Nanjing Medical University Changzhou 213000 China; 5 Orthopedics and Sports Medicine Center Suzhou Municipal Hospital Nanjing Medical University Affiliated Suzhou Hospital Suzhou 215000 China

**Keywords:** synovial inflammation, macrophage polarization, κ-opioid receptor, osteoarthritis, NF-κB

## Abstract

Osteoarthritis (OA) is a prevalent and chronic joint disease that affects the aging population, causing pain and disability. Macrophages in synovium are important mediators of synovial inflammatory activity and pathological joint pain. Previous studies have demonstrated the significant involvement of κ-opioid receptor (KOR) in the regulation of pain and inflammation. Our study reveals a significant reduction in synovial KOR expression among patients and mice with OA. Here, we find that KOR activation effectively inhibits the expressions of the LPS-induced-inflammatory cytokines TNF-α and IL-6 by inhibiting macrophage M1 phenotype. Mechanistically, KOR activation effectively suppresses the proinflammatory factor secretion of macrophages by inhibiting the translocation of NF-κB into the nucleus. Our animal experiments reveal that activation of KOR effectively alleviates knee pain and prevents synovitis progression in OA mice. Consistently, KOR administration suppresses the expressions of M1 macrophage markers and the NF-κB pathway in the synovium of the knee. Collectively, our study suggests that targeting KOR may be a viable strategy for treating OA by inhibiting synovitis and improving joint pain in affected patients.

## Introduction

Recent advancements in imaging techniques have enabled the characterization of the osteoarthritis (OA) phenotype, revealing pathological changes such as cartilage degeneration, subchondral osteosclerosis and synovial inflammation [1]. The primary symptoms experienced by patients include gradually worsening pain in the knee, accompanied by swelling, stiffness and reduced mobility [2]. In severe cases, these symptoms can result in joint deformity or complete loss of joint function, greatly impacting the patient’s ability to perform daily activities and work. Despite the availability of current pain relief medications for OA, their effectiveness can be inadequate and with potentially serious side effects [ 3, 4]. Therefore, it is important to explore new and improved treatments for OA.

The synovium undergoes significant changes during the progression of OA disease, even before cartilage degeneration occurs [5]. These changes are mainly characterized by monocyte infiltration and the production of inflammatory cytokines in the synovium lining. A previous report indicated that synovitis is present in almost all stages of OA pathogenesis and is linked to pain and poor function in patients [6]. Therefore, targeting key aspects of synovial inflammation is a promising approach for both pain relief and structural modification. Recent studies have demonstrated the involvement of synovial inflammation in the early stages of OA, particularly in the activation of the innate inflammatory pathway [ 7, 8]. This pathway serves as a central mediator in the pathogenesis of OA and contributes significantly to its progression. This innate inflammatory pathway is triggered by damage-associated molecular patterns (DAMPs) which primarily originate from protease-mediated persistent destruction of cartilage and meniscus tissue, as well as tissue damage caused by mechanical stimulation [9].

Macrophages play crucial roles in synovial inflammatory activity and pathological OA injury, and are essential cells of the innate immune response [10]. In an *in vivo* imaging study, it was found that the number of activated macrophages in the knee joint is closely associated with the severity and symptoms of radiologic OA [11]. Moreover, further investigations have demonstrated that macrophages are responsible for OA pain, and the measurement of soluble macrophage markers can accurately predict the risk of OA progression [ 12, 13]. In the context of OA, macrophages are divided into two polarized states: M1 and M2. M1 macrophages are associated with proinflammatory cytokines such as tumor necrosis factor (TNF), interleukin (IL)-1β, and IL-6, which stimulate catabolic mediators like metallomatrix proteinases in both synovium and cartilage [ 14, 15]. In order to alleviate the immune microenvironment of synovial tissue and improve the progression of OA disease, it may be beneficial to regulate synovial macrophage polarization.

Opioids have been used in the clinical treatment of diseases for many years, and the analgesic effects of opioids exert biological effects through opioid receptors [16]. Classical opioid receptors mainly include μ-opioid receptors (Mu opioid receptors, MORs), δ-opioid receptors (Delta opioid receptors, DORs), and κ-opioid receptors (Kappa opioid receptors, KORs) [17]. KOR is a member of the rhodopsin subclass of the G protein-coupled receptor (GPCR) superfamily and is involved in the treatment of various diseases. Recent studies have revealed that activation of KOR is effective in treating pruritus, multiple sclerosis, Alzheimer’s disease, cancer, and local ischemia [ 18– 20]. Bileviciute-Ljungar *et al*. [21] found that activation of KOR reduced ankle inflammation and cartilage damage in a rat arthritis model. According to Wu *et al*. [22], when KOR is absent in mice, cartilage degeneration levels increase after injury due to increased expressions of catabolic enzymes and proinflammatory cytokines. Weber *et al*. [23] reported that the KOR agonist JT 09 supported a novel molecular mechanism of KOR action in osteoarthritis by regulating Hedgehog signaling and thereby reducing articular chondrocyte and cartilage explant matrix degeneration in a rat *in vivo* model.

Nuclear factor-κB (NF-κB) is a group of transcription factors that can be activated by various proinflammatory cytokines. NF-κB plays a crucial role in inflammation and differentiation of mammalian cells [24]. Inflammatory signals activate IκB kinases, which results in the degradation of IκB. Subsequently, the NF-κB complex is translocated to the nucleus, where it initiates the transcription of target genes [25]. NF-κB signaling is extensively involved in the pathology of OA through multiple mechanisms [26]. Previous studies have reported that spermidine mitigates the progression of OA by deubiquitinating RIP1 in synoviocytes and inhibiting the NF-κB pathway induced by TNF-α [27]. In addition, a link between KOR and NF-κB signaling has been discovered. Lin *et al*. [28] found that KOR stimulation affects the heart of rats with ischemia-reperfusion through the TLR4/NF-κB signaling pathway. Similarly, Zhou *et al*. [29] reported that activating KOR showed positive effects on vascular dysfunction in streptozotocin-induced diabetic rats. In hence, the KOR-mediated NF-κB cascade may play a role in the progression of synovitis in OA.

However, it remains unclear whether KOR can mitigate OA synovitis. Our study revealed a down-regulation of KOR expression in the synovial tissues of knee joints in OA patients, which was found to be negatively correlated with the level of tissue inflammation. We used LPS intervention in RAW264.7 cells to mimic the activation of the inflammatory microenvironment in knee macrophages. Furthermore, we investigated the effects of the KOR agonist U50488H on macrophage inflammation and synovitis *in vitro* and *in vivo*. The objective of this study was to explore a new promising approach for the treatment of OA.

## Materials and Methods

### Cell culture

RAW264.7 macrophages from the American Type Culture Collection (ATCC; Manassas, USA) were grown in Dulbecco’s modified Eagle medium (DMEM) containing 100 U/mL penicillin, 100 μg/mL streptomycin sulfate (Lifetech, New York, USA) and 10% fetal bovine serum (Gibco, New York, USA). Cell Counting Kit-8 reagent was purchased from NCM Biotech (Suzhou, China). LPS was purchased from Solarbio (Beijing, China). MIA was acquired from Sigma (Gillingham, UK). U50488H, a KOR agonist and neferine were acquired from MedChemExpress (Monmouth Junction, USA).

### Cell proliferation assay

To measure cell viability, the CCK-8 cell viability assay was used on RAW264.7 macrophages treated with varying concentrations (0, 2, 4, 8, 16, 32, and 64 μM) of U50488H for 24, 48, and 72 h. After incubation with 10 μL of CCK-8 solution, absorbance was measured at 450 nm using an automated porous spectrophotometer (Molecular Device, Sunnyvale, USA) after 2 h.

### Western blot analysis

In this study, RIPA lysis buffer was added to RAW 264.7 macrophages and incubated on ice for 30 min. The lysate was then centrifuged at 4°C for 20 min and the supernatant was collected to determine the protein concentration by using a BCA kit (Beyotime, Shanghai, China). The proteins were then denatured by boiling at 95°C for 8 min, separated by SDS-PAGE, and transferred onto PVDF membranes (Millipore, Billerica, USA). Membranes were cut horizontally and blocked with 5% skim milk for an hour at room temperature. Primary antibodies, including anti-KOR (1:1000; Abclonal, Wuhan, China), anti-iNOS (1:1000; ProteinTech, Chigaco, USA), anti-Arg1 (1:5000; ProteinTech), anti-TNF-α (1:1000; ABclonal), anti-IL-6 (1:1000; ABclonal), anti-P-P65 (1:1000; ABclonal), anti-P65 (1:1000; ABclonal), anti-P-IKBα (1:500; ABclonal), anti-IKBα (1:500; ABclonal) and anti-β-actin (1:5000; ProteinTech), were added and incubated overnight at 4°C. The following day, the membranes were rinsed three times with 0.3% TBST and incubated with the HRP-conjugated secondary antibodies (1:5000; Abcam, Cambridge, UK) for 1 h at room temperature. The washed PVDF membranes were submerged in a chemiluminescence developer solution. The intensity of the resulting bands was determined by densitometric analysis using ImageJ software (Media Cybernetics, Bethesda, USA). The results were normalized to the densitometric value of β-actin.

### Immunofluorescence staining

RAW264.7 macrophages were seeded in 24-well culture plates, and cell climbing sections were placed inside the culture plates for immunofluorescence detection. Immunofluorescence staining was performed using anti-iNOS (1:200; ProteinTech), anti-Arg-1 (1:200; ProteinTech), anti-KOR (1:200; Abclonal) and anti-P-P65 (1:200; ABclonal) antibodies diluted in 1% BSA. After primary antibody incubation overnight at 4°C, the primary antibody was aspirated and rinsed with PBS solution for 15 min. The fluorescent secondary antibody (AlexaFluor® 488; Abcam) diluted in 1% BSA was used to incubate with the samples for 2 h at room temperature. After the samples were rinsed with PBS for 15 min, images were observed and photographed with a confocal microscope (Zeiss, Wetzlar, Germany), and fluorescence intensity was determined with ImageJ software.

### Flow cytometry

Cells were treated with various methods and then fixed with 4% paraformaldehyde for 15 min. Then, the samples were stained with FITC-conjugated anti-inducible nitric oxide synthase (iNOS; BD Biosciences, Franklin Lakes, USA) and PE-conjugated anti-arginase 1 (Arg1; R&D Systems, Minneapolis, USA) at 4°C in the dark for 30 min. After extensive wash, PBS was added to adjust the cell concentration. Cells were analyzed with a CytoFLEX flow cytometer (Beckman, Pasadena, USA), and data were analyzed using FlowJo (Ashland, USA).

### Management of the synovial tissue in OA patients

All human experiments conducted in this study were in compliance with the ethical standards of Suzhou Hospital Affiliated to Nanjing Medical University. Synovial tissues were obtained from two groups of patients: those who underwent total knee replacement due to knee OA (OA group, *n*=5, 3 females and 2 males; median age, 66 years; age range, 60–74 years) and those who underwent knee arthroscopic surgery due to anterior cruciate ligament injury (non-OA group, *n*=5, 2 females and 3 males; median age, 38 years; age range, 32‒45 years). The synovial tissue was surgically removed and cut into uniformly sized pieces. These pieces were again washed three times with PBS before being embedded and cut into blocks for further staining.

### Animal experiments

Animal experiments were conducted following the ethical guidelines of Suzhou Hospital affiliated to Nanjing Medical University. C57BL mice (aged 6‒8 weeks; Suzhou Sinosai Biotechnology, Suzhou, China) were housed under standard laboratory conditions at a temperature range of 22‒26°C. The mice were randomly assigned to three groups, with each group consisting of 7 mice: sham group, MIA-OA group, and U50488H group. To prepare knee OA models, the previous method [30] was followed for the MIA-OA and U50488H groups. All mice were fasted for half a day and injected intraperitoneally under general anesthesia. The mice were fixed, the skin was prepared and sterilized, and each mouse was injected with 0.1 mg MIA into the left hind leg knee in a volume of 10 μL. During the 21-day period, the mice were given unrestricted access to water and allowed to move freely. After this period, the U50488H group was intraperitoneally given U50488H (1.5 mg/kg) for 7 days, while the sham and MIA-OA groups were given equal doses of saline.

### Mechanical pain threshold

To test the mechanical pain sensitivity, the Von Frey method [31] was employed. The mice were placed in a metal mesh cage, with the upper part fixed in the cage frame and the lower part suspended for easy observation. Prior to testing, mice were acclimated to their home cage for 1 h. During testing, von-Frey cilia with varying folding forces (0.4, 0.6, 1.0, 1.4, 2.0, and 4.0 g) were used to stimulate the position between the bones of the plantar foot, starting with the smallest force and progressing to the largest. The duration of the stimulation was 8 s; if the mice showed positive responses such as foot contraction or foot licking during this time, the same cilia stimulation was repeated for 30 s with an interval of 5 min between different cilia stimulations. Five positive responses were required for the same cilia stimulation to determine the minimum stimulation intensity that elicited a foot contraction response threshold for mechanical stimulation. If there was still no traction response after cilia stimulation, the maximum value was recorded.

### Thermal pain threshold

The Hargreaves method [32] was used to determine the withdrawal latency (PWL) to heat pain in mice. The mice were placed on a heated glass plate inside a metal mesh cage and a light was directed onto the sole surface of the right hind paw. The intensity of the light was gradually increased until the mouse withdrew its paw from the heat source, which marked the onset of thermal stimulation. The time from the onset of thermal stimulation to paw removal (in seconds) corresponds to the withdrawal latency of the paw. Four latency values were obtained in the right hind paw. An interval of 15 s was set to prevent tissue heat source damage.

### Histopathology

Mice were sacrificed on day 28, and the freshly obtained whole left posterior knee joint was fixed in 4% paraformaldehyde for 2 days. Then, the tissue was placed in 10% ethylenediaminetetraacetic acid (EDTA; Sigma) and embedded in paraffin for 15 days. Mouse joint samples were sagitally sliced (6 μm thick) for hematoxylin-eosin (H&E) staining and immunohistochemistry (IHC) staining, and images were acquired under an axial 40C optical microscope (Zeiss). The severity of synovitis was assessed by examining the enlargement of the synovial lining cell layer, density of resident cells, and inflammatory infiltrate. The positive cell number (brown gray) of the section was utilized to evaluate the IHC staining.

### Statistical analysis

Data were analyzed using GraphPad Prism software (version 8.0) and presented as the mean±standard deviation (SD). A Student’s *t-*test was conducted to compare mean values between two groups, while one-way ANOVA was used for comparisons among more than two groups. Pairwise comparisons between multiple groups were performed using Tukey-test. *P*<0.05 was considered statistically significant.

## Results

### The expression of KOR was downregulated in patients and mice with OA

**Figure 1:**
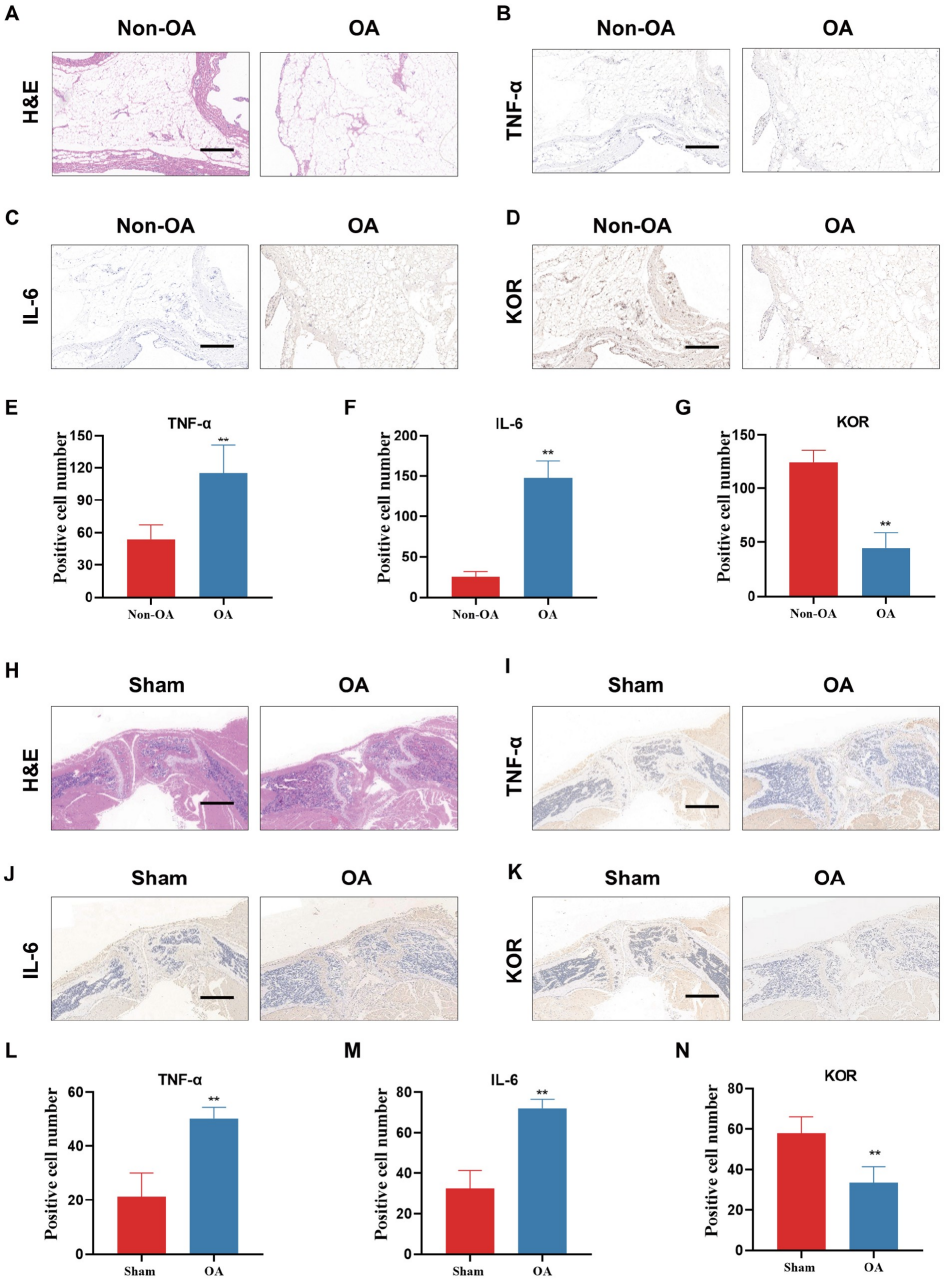
Figure 1 The expression of KOR was downregulated in patients and mice with OA (A) H&E staining of synovial tissues in ACL injury and OA patients. (B‒D) TNF-α, IL-6 and KOR IHC staining of synovial tissues in ACL injury and OA patients. (E‒G) Quantification of IHC staining for TNF-α, IL-6 and KOR. (H) H&E staining of synovial tissues in sham and OA mice. (I‒K) TNF-α, IL-6 and KOR IHC staining of synovial tissues in sham and OA mice. (L‒N) Quantification of IHC staining for TNF-α, IL-6 and KOR. Scale bar: 100 μm. *P<0.05; **P<0.01.

To explore whether KOR plays a role in the development and progression of OA, we first examined the expression of KOR in the synovium of human OA. To serve as the non-OA group, we opted to use synovium with anterior cruciate ligament (ACL) injury. H&E staining showed that patients with OA had poorer synovial histology and more severe inflammatory infiltration ( Figure 1A). Immunohistochemical (IHC) staining showed that synovial patients with osteoarthritis undergoing total knee arthroplasty had more severe inflammation, higher expressions of inflammatory factors and decreased expression of KOR compared with patients with ACL injury ( Figure 1B‒G). We then analyzed KOR expression during OA progression using a MIA-induced OA mouse model. Similarly, in OA mice, the synovium exhibited increased levels of inflammatory infiltration and expressed higher levels of inflammatory factors TNF-α and IL-6 ( Figure 1H‒J,L‒M). The expression of KOR was downregulated in the synovium of OA mice compared to normal mice ( Figure 1K,N). Collectively, these results indicated that KOR expression was downregulated as OA progressed, suggesting a potential role of KOR in OA pathogenesis.

### Activation of KOR resulted in the inhibition of inflammatory factor expression

**Figure 2:**
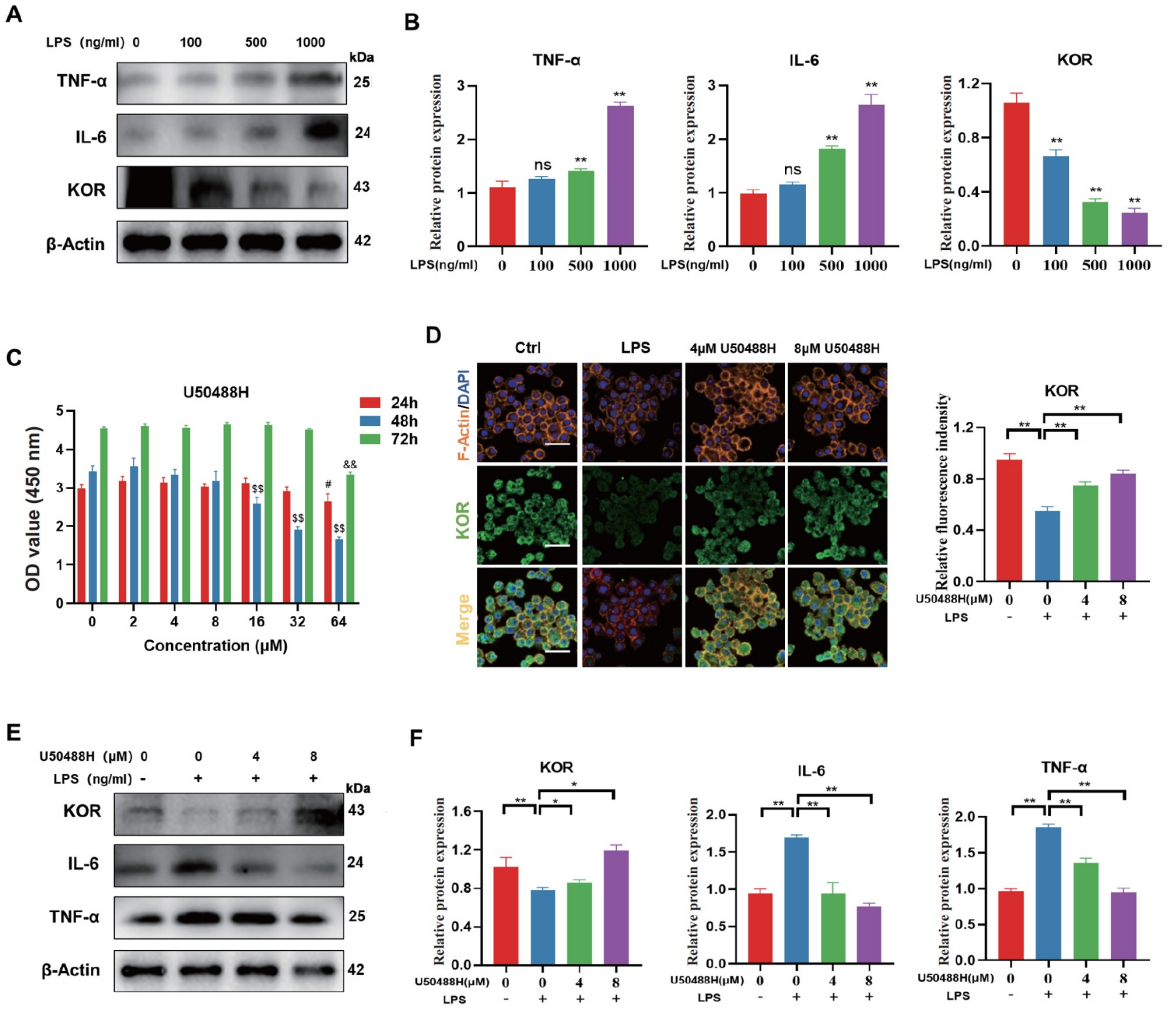
Figure 2 Activation of KOR resulted in the inhibition of inflammatory factor expression (A,B) Western blots and quantitative analysis showing the expression levels of TNF-α, IL-6 and KOR after treatment with different concentrations of LPS. (C) CCK-8 results showing the cytotoxicity of U50488H on RAW264.7 cells. The concentrations of U50488H were compared with 0 μmol/mL at 24 h (#P<0.05), 48 h ($P<0.05), and 72 h (&P<0.05). (D) Immunofluorescence staining of KOR in RAW264.7 cells. (E,F). Western blots and quantitative analysis showing the expression levels of TNF-α, IL-6 and KOR after treatment with U50488H. *P<0.05; **P<0.01.

To mimic the OA inflammatory microenvironment, we utilized RAW264.7 cells treated with varying concentrations of LPS (100, 500, and 1000 ng/mL) for 24 h to assess the expressions of KOR and inflammatory factors. Our findings indicated that as the LPS concentration increased, the expressions of inflammatory factors were also increased. Conversely, the expression of KOR was gradually downregulated ( Figure 2A,B). Our hypothesis is that KOR expression may be linked to inflammation in the LPS-induced inflammatory environment of RAW264.7 cells.

To investigate the involvement of KOR in the inflammatory changes in LPS-treated RAW264.7 cells, we treated RAW264.7 cells with a KOR specific agonist, U50488H. We first conducted a CCK-8 assay to determine the optimal concentration of U50488H. We detected the effects of different concentrations of U50488H on RAW activity at 24, 48, and 72 h. The results indicated that the RAW activity was the highest when the concentration of U50488H was less than 8 μM ( Figure 2C). Subsequently, we treated LPS-stimulated RAW264.7 cells with 4 μM and 8 μM U50488H for 24 h, respectively, to detect the protein expression levels of KOR and inflammatory factors. The immunofluorescence results indicated that U50488H promoted KOR expression in a concentration-dependent manner ( Figure 2D). Western blot analysis results also showed that the expression levels of proinflammatory cytokines IL-6 and TNF-α were gradually downregulated with increasing concentrations of U50488H ( Figure 2E,F). Therefore, it is possible that KOR could alleviate the inflammatory microenvironment caused by LPS-stimulated RAW264.7 cells.

### Activation of KOR attenuated macrophage M1 polarization in LPS-stimulated RAW264.7 cells

Macrophage activation is crucial for maintaining tissue homeostasis, regulating immunity, and influencing disease progression [ 33, 34]. Different factors can determine macrophage activation states and phenotypes, resulting in two distinct subtypes: proinflammatory M1 macrophages and anti-inflammatory M2 macrophages [35]. Macrophage polarization plays a crucial role in various pathological processes, including the mediation of inflammation. To investigate whether KOR regulates inflammation by modulating macrophage polarization in LPS-stimulated RAW264.7 cells, we examined the changes in M1 and M2 macrophages following KOR excitation by U50488H.

**Figure 3:**
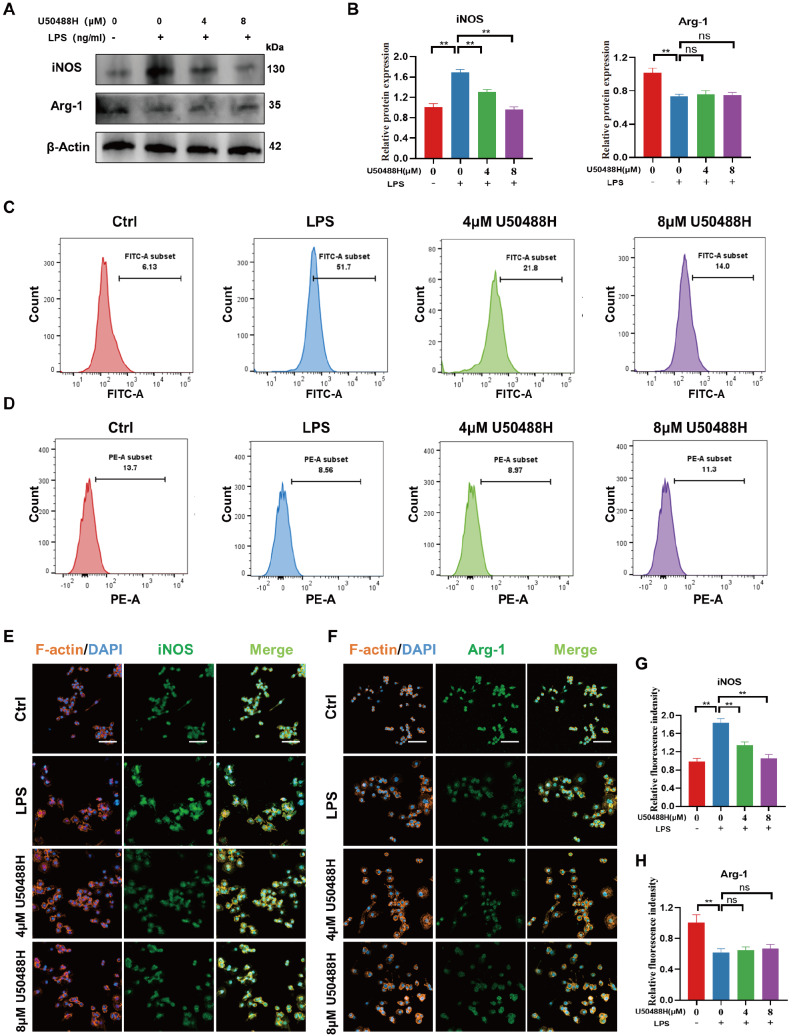
Figure 3 Activation of KOR attenuated macrophage M1 polarization in LPS-stimulated RAW264.7 (A,B) Western blots and quantitative analysis showing the expression levels of iNOS and Arg-1 after treatment with U50488H. (C,D) Flow cytometric analysis of iNOS and Arg-1 in LPS-stimulated RAW264.7 cells after treatment with U50488H. (E,F) Immunofluorescence staining of iNOS and Arg-1 in LPS-stimulated RAW264.7 cells after treatment with U50488H. Scale bar: 50 μm. (G,H) Quantitative analysis of immunofluorescence staining for iNOS and Arg-1. *P<0.05; **P<0.01.

Firstly, western blot analysis results revealed that RAW264.7 cells treated with LPS showed increased iNOS levels compared to the control group, indicating a higher proportion of M1-type macrophages. However, treatment with U50488H resulted in a concentration-dependent decrease in iNOS levels, suggesting a decrease in M1-type macrophages. Notably, the drug did not have a significant effect on the expression of M2 macrophage marker Arg-1 ( Figure 3A,B). Furthermore, flow cytometry was used to measure the expression levels of M1 and M2 macrophage markers. The results indicated that activation of KOR led to a significant decrease in iNOS expression, with little effect on the level of Arg-1 ( Figure 3C,D). This finding is consistent with the results obtained from immunofluorescence staining ( Figure 3E‒H). In conclusion, KOR can reduce the M1-type macrophage polarization of RAW264.7 cells induced by LPS, but it has little effect on M2 polarization.

### Activation of KOR inhibited M1 polarization via the NF-κB signaling pathway

**Figure 4:**
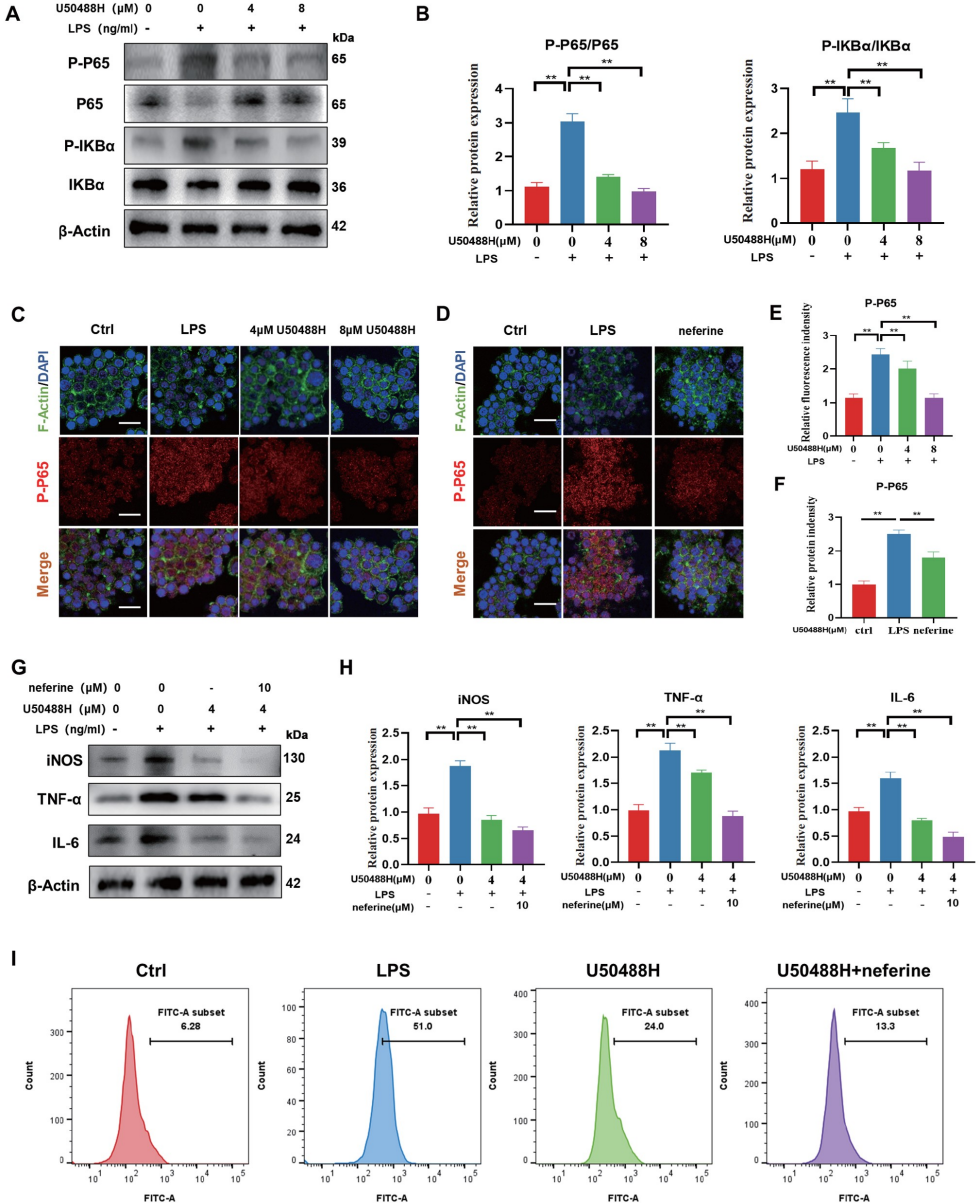
Figure 4 Activation of KOR inhibited M1 polarization via the NF-κB signaling pathway (A) Western blots showing the expression levels of P-P65, P65, P-IKBα and IKBα after treatment with U50488H. (B) Quantitative analysis of P-P65/P65 and P-IKBα/IKBα. (C) Immunofluorescence staining of P-P65 in LPS-stimulated RAW264.7 cells after treatment with U50488H. (D) Immunofluorescence staining of P-P65 in LPS-stimulated RAW264.7 cells after treatment with neferine. Scale bar: 50 μm. (E) Quantitative analysis of P-P65 after treatment with U50488H. (F) Quantitative analysis of P-P65 after treatment with neferine. (G,H) Western blots and quantitative analysis showing the expression levels of iNOS, TNF-α and IL-6. (I) Flow cytometry of iNOS in LPS-stimulated RAW264.7 cells after treatment with U50488H and neferine. *P<0.05; **P<0.01.

The pro-inflammatory pathway NF-κB is known to play a role in macrophage polarization towards the pro-inflammatory M1 type [ 36, 37]. To investigate the relationship between KOR activation and inhibition of M1 polarization, we examined targeted proteins in the NF-κB signaling pathway. Our findings revealed that LPS stimulation led to a significant increase in phosphorylation levels of P65 and IKBα. However, treatment with U50488H inhibited this effect ( Figure 4A,B). Furthermore, immunofluorescence staining results showed that P65 shifted from the cytoplasm to the nucleus upon LPS stimulation, and activation of KOR could reverse this trend ( Figure 4C,E). Subsequently, we explored whether neferine, an inhibitor of NF-κB pathway, contributes to this pathway. Immunofluorescence staining showed that the addition of neferine significantly reduced the nuclear entry of P-P65 following LPS stimulation ( Figure 4D,F). We utilized 10 μM neferine to coculture cells with low doses of U50488H. Western blot analysis results indicated that neferine enhanced the inhibitory effect of U50488H on inflammation and M1 macrophage polarization ( Figure 4G,H). Additionally, our flow cytometry analysis revealed that U50488H was able to inhibit the M1 macrophage ratio. However, this effect was further enhanced when combined with neferine ( Figure 4I). Collectively, these findings suggest that the activation of KOR may regulate the polarization of M1-type macrophages through the NF-κB signaling pathway.

### Activation of KOR ameliorated painful behaviors and synovial inflammation in OA mice

**Figure 5:**
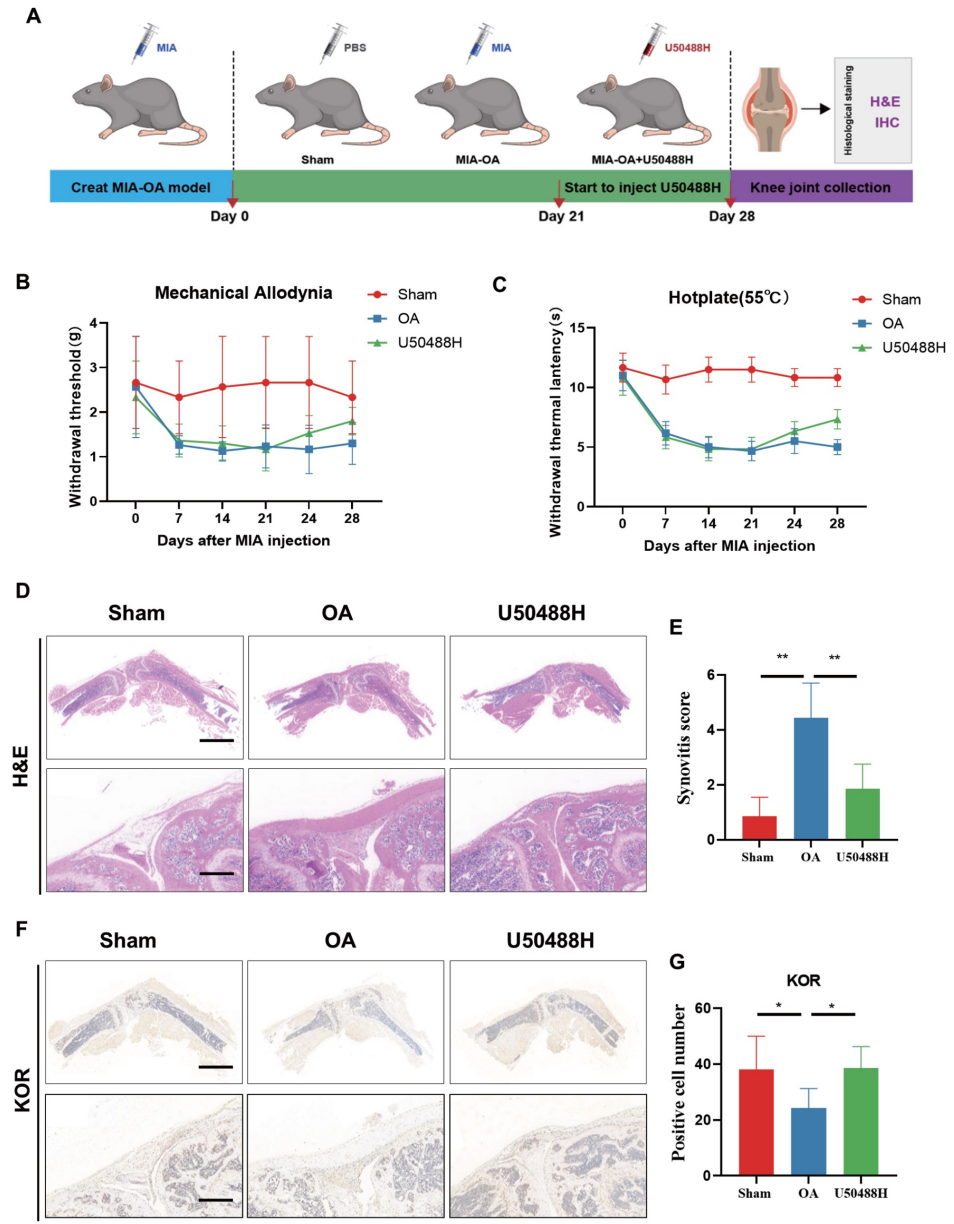
Figure 5 Activation of KOR ameliorated painful behaviors and synovial inflammation in OA mice (A) Schematic diagram of the in vivo experiments. (B) Mechanical pain measurement. (C) Thermal pain measurement. (D) H&E staining of synovial tissues in OA mice. (E) Synovitis score. (F,G) KOR IHC staining and quantitative analysis of synovial tissues in OA mice. Scale bar: 100 μm. *P<0.05; **P<0.01.

Previous *in vitro* experiments have demonstrated the significant anti-inflammatory benefits of U50488H. In order to further understand its effects, we conducted *in vivo* experiments to investigate the potential benefits of U50488H. To create a mouse model of OA, we injected MIA into the knee joint of mice. The experimental procedure is depicted in Figure 5A. Mechanical and thermal pain results showed that both mechanical and thermal pain thresholds were reduced in the MIA-OA group mice compared to the sham group mice, lasting from day 7 to day 21. After 7 days of continuous injection of U50488H, both mechanical and thermal pain thresholds were elevated in mice compared to those of the MIA-OA group, which indicated that activation of KOR could reduce the pain behavior of OA mice ( Figure 5B,C). Furthermore, H&E staining of knee sections of mice revealed that the agonist-injected mice had reduced synovial inflammation, decreased synovial thickening, reduced inflammatory cell infiltration, and significantly lower synovitis scores compared with OA mice ( Figure 5D‒E). Subsequently, immunohistochemical staining was used to detect the expression of KOR in mouse synovium. The results indicated that KOR expression in the synovium of OA mice was lower than that in the sham group. However, after U50488 intervention, KOR expression in mouse synovitis was significantly increased ( Figure 5F‒G). This suggests that targeting KOR in synovitis may be a potential therapeutic option for suppressing synovitis.

### Activation of KOR inhibited M1 polarization and NF-κB signaling in OA mice synovium

**Figure 6:**
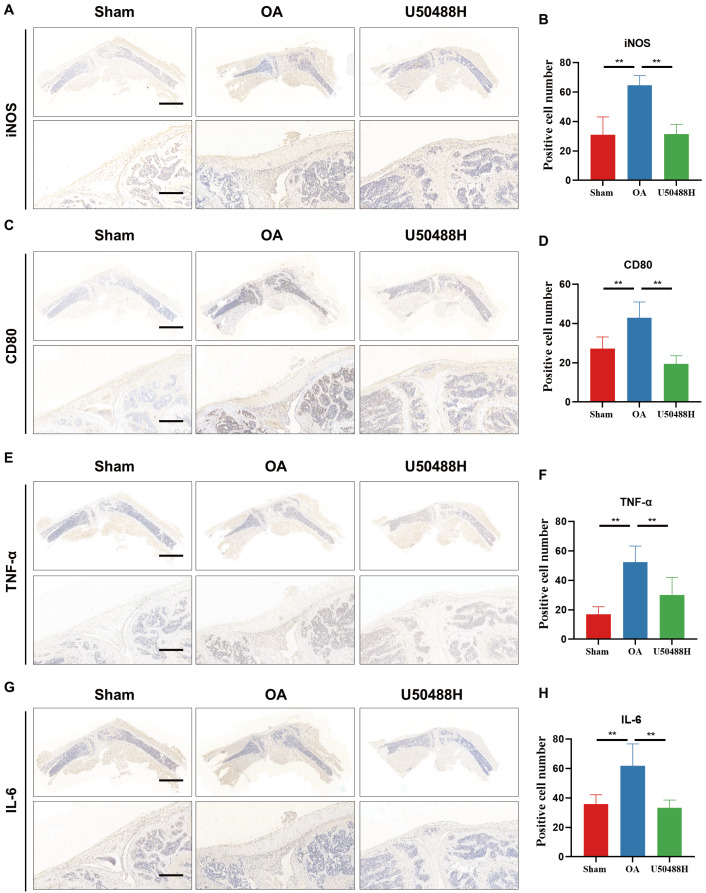
Figure 6 Activation of KOR inhibited M1 polarization in OA mice synovium (A,B) iNOS IHC staining and quantitative analysis of synovial tissues in OA mice. (C,D) CD80 IHC staining and quantitative analysis of synovial tissues in OA mice. (E,F) TNF-α IHC staining and quantitative analysis of synovial tissues in OA mice. (G,H) IL-6 IHC staining and quantitative analysis of synovial tissues in OA mice. Scale bar: 100 μm. *P<0.05; **P<0.01.

Meanwhile, we further investigated the expression levels of macrophage markers in the synovial of mice, and immunohistochemical staining revealed that the expressions of M1 macrophage markers iNOS and CD80 were significantly suppressed in MIA-OA mice after U50488H treatment ( Figure 6A‒D). In addition, immunohistochemical results also showed that the injection of U50488H reduced the expression levels of synovial proinflammatory factors TNF-α and IL-6, all of which indicated that KOR activation could effectively reduce OA in mice ( Figure 6E‒H). In addition, this could suggest that our activation of KOR may alleviate OA in mice by inhibiting M1-type macrophage polarization.

**Figure 7:**
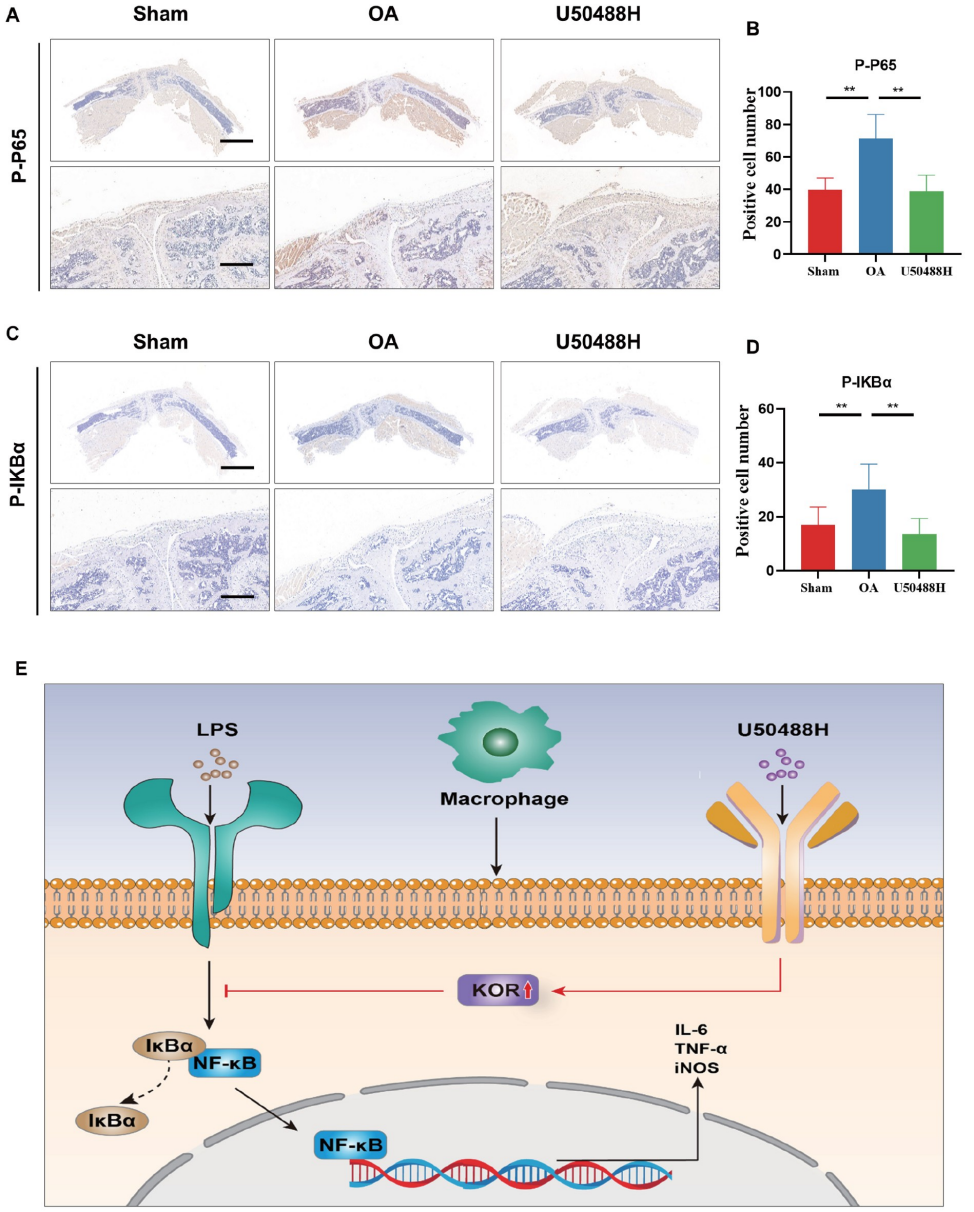
Figure 7 Activation of KOR inhibited the NF-κB signaling pathway in OA mice synovium (A,B) P-P65 IHC staining and quantitative analysis of synovial tissues in OA mice. (C,D) P-IKBα IHC staining and quantitative analysis of synovial tissues in OA mice. (E) Schematic diagram of U50488H acting on the synovium. Scale bar: 100 μm. *P<0.05; **P<0.01.

To investigate whether U50488H’s inflammation-suppressing benefits *in vivo* are linked with NF-κB signaling, we performed immunohistochemical staining of the NF-κB pathway phosphoprotein P65 and IKBα. We found significant upregulation of P-P65 and P-IKBa expressions in the synovium of OA mice compared to that in the sham group. However, following the injection of U50488H into the articular cavity, there was a significant downregulation of P-P65 and P-IKBa expressions in the synovial tissue of OA mice, which were consistent with our *in vitro* study ( Figure 7A‒D). These results suggest that activation of KOR may regulate the polarization of M1-type macrophages via the NF-κB signaling pathway, alleviating pain behavior and synovial inflammation in mice ( Figure 7E).

## Discussion

OA is a degenerative disease that is prevalent among elderly individuals. It affects over 500 million people worldwide as of 2020, and its incidence increases with age [ 38, 39]. OA significantly impacts the quality of life of older patients and is the leading cause of disability in this population. The condition initially causes damage to cartilage, which then progresses to joint space narrowing, synovial inflammation, subchondral bone sclerosis, and osteophyte formation [ 40‒ 42]. Clinically, it is characterized by joint pain, deformity, and dysfunction. Studies have shown that the synovial membrane undergoes an inflammatory process in OA, with a significant increase in the expression of matrix metalloproteinases (MMPs) and the secretion of proinflammatory cytokines [43].

Synovial inflammation is the most typical manifestation of OA inflammation [ 6, 44]. Macrophages are one of the most abundant immune cells in the synovium, and play a key role in maintaining the homeostasis of healthy synovial tissue [35]. Macrophages are cellular mediators of innate immunity and tissue remodeling that play a crucial role in host defense and physiological homeostasis [45]. The intrasynovial inflammatory microenvironment is composed of an inflammatory infiltrate consisting of synovial macrophages. These macrophages are believed to be a significant contributor to the development of OA. Macrophages are present in large quantities within the synovium and play a crucial role in maintaining a healthy synovial environment [ 45, 46]. The presence of an accumulation of polarized macrophages in the endothelial lining is a notable visual indicator of synovitis. Macrophage polarization is not only increasingly widely used in other diseases, but also a research hotspot to explore the treatment of osteoarthritis. At present, there are many studies on the pathogenesis of OA, and a large number of studies have shown that macrophages play an important role in OA [ 47, 48].

Increasing evidence suggests that M1/M2 macrophage polarization imbalance plays an important role in OA inflammation [ 49, 50]. In the course of OA progression, synovial macrophages are activated, accumulated, and differentiated into two distinct forms, namely, M1 and M2 macrophages [51]. M1 macrophages are stimulated by interferon, LPS, or TNF-α and produce a great number of pro-inflammatory cytokines and mediators, for example, TNF-α, IL-1β, IL-6, IL-12 and cyclooxygenase-2 (COX-2) [52]. On the other hand, M2 macrophages, often referred to as wound-healing macrophages, are initiated by IL-4 and IL-13 and are involved in tissue healing and reconstruction [53]. Liu *et al*. [54] found that the proportion of M1/M2 macrophages in synovial fluid and peripheral blood was significantly higher in knee OA patients compared to healthy controls. According to Hang *et al*. [46], promoting macrophage polarization to M1 type worsened OA by activating β-catenin signaling in chondrocytes and through the secretion of Rspo2. Sun *et al*. [55] found that blocking TRPV 4 inhibited M1 macrophage polarization through ROS/NLRP3 signaling, thereby improving OA. Consequently, it is essential to investigate macrophage alteration to reduce M1-type polarization for the management of osteoarthritis. Inhibition of M1 polarization of synovial macrophages can alleviate the synovial inflammatory microenvironment, which is a promising therapeutic strategy for OA.

KOR is a member of the G protein-coupled receptor family that plays a crucial role in pain management and relief [56]. Recent studies have indicated that KOR has the potential to regulate macrophage polarization effectively. In particular, research suggests that KOR can reduce LPS-induced acute lung injury by inhibiting the polarization of M1 macrophages while promoting the polarization of M2 macrophages [57]. When KOR is activated, it activates the extracellular signal-regulated kinase (ERK) pathway and the phosphatidylinositol 3-kinase (PI3K) pathway [58]. By activating these pathways, KOR can reduce the levels of these inflammatory mediators, thus decreasing inflammation. In our study, we revealed that the expression of KOR is reduced in OA patients and mice, indicating that the absence of KOR may worsen the onset of OA. To further investigate this, we conducted *in vitro* experiments by treating RAW264.7 cells with LPS to simulate pathological OA. Our findings showed that the addition of the KOR agonist U50488H not only reduced the expressions of inflammatory cytokines like IL-6 and TNF-α, but also inhibited the polarization of M1 macrophages. The results were further confirmed by animal experiments *in vivo*.

OA is a condition that is marked by low-level, long-term inflammation. This inflammation is a key factor in the development and worsening of OA, and the NF-κB signaling pathway has been identified as a critical player in this inflammatory response [59]. The NF-κB signaling pathway is activated by several ligands, including TNF-α, IL-1β, and LPS, leading to the phosphorylation of IκB and proteasomal degradation [60]. The proinflammatory cascade is closely associated with M1 macrophages, and inhibition of NF-κB signaling plays a central role in OA treatment by controlling the inflammatory response in macrophages. Lu *et al*. [10] showed that HA significantly reduced M1 type macrophages and attenuated synovitis via inhibition of P65/NF-κB signaling, thereby improving OA. Zheng *et al*. [61] found that flavin reduced NO production of IL-1β by inhibiting the NF- κB pathway activation in the human OA synovium. As a classical proinflammatory pathway, NF-κB has been extensively studied in macrophage polarization in OA synovium. In this study, we found that the polarization of inhibitory M1 macrophages after KOR activation was mediated through the NF-κB pathway, and that U50488H alleviated the inflammatory microenvironment and M1 macrophage polarization through inhibition of NF-κB activation. The combination of the NF-κB inhibitor neferine and U50488H has been shown to have a greater ability to inhibit inflammation and macrophage M1 phenotype compared to using U50488H alone.

Nevertheless, there are some limitations in our study. We did not include gene knockout mice which could have helped us in better understanding the role of KOR in controlling synovial macrophage polarization. Additionally, our experiments were conducted on male C57 mice in an MIA-OA model. Further exploration is necessary to determine the contribution of KOR to OA pain in elderly mice.

In conclusion, we demonstrated that KOR is a key target for mediating the inflammatory microenvironment and macrophage polarization during the pathogenesis and progression of OA. Our research revealed that as OA progresses, there is a notable decrease in KOR expression, which may in turn exacerbates synovial inflammation. The secretion of proinflammatory factors and the conversion of M1 macrophages are effectively inhibited by the administration of KOR agonists, which ultimately leads to the reduction of synovitis. This process may be influenced by the deactivation of the NF-κB signaling pathway. Taken together, our findings suggest that activating KOR may be a significant treatment for OA, and that KOR agonists may alleviate the inflammatory microenvironment and thereby treat OA.
